# Spectrum of BRCA1 interacting helicase 1 aberrations and potential prognostic and therapeutic implication: a pan cancer analysis

**DOI:** 10.1038/s41598-023-31109-6

**Published:** 2023-03-17

**Authors:** Guo Long, Kuan Hu, Xiaofang Zhang, Ledu Zhou, Juanni Li

**Affiliations:** 1 Department of Hepatobiliary Surgery, Xiangya Hospital, Central South University, Changsha, 410008 Hunan China; 2 Departments of Burn and Plastic, Ningxiang People’s Hospital, Hunan University of Chinese Medicine, Changsha, 410600 Hunan China; 3 Department of Pathology, Xiangya Hospital, Central South University, Changsha, 410008 Hunan China; 4 National Clinical Research Center for Geriatric Disorders, Xiangya Hospital, Central South University, Changsha, 410008 Hunan China

**Keywords:** Cancer genetics, Targeted therapies

## Abstract

BRCA1 interacting helicase 1 (BRIP1) alteration was crucial in tumors and it was a potential therapeutic target in ovarian serous cystadenocarcinoma (OV). Although a small number of studies had focused on BRIP1, an extensive study of BRIP1 genetic mutation and its clinical application in different cancer types had not been analyzed. In the current study, we analyzed BRIP1 abnormal expression, methylation, mutation, and their clinical application via several extensive datasets, which covered over 10,000 tumor samples across more than 30 cancer types. The total mutation rate of BRIP1 was rare in pan cancer. Its alteration frequency, oncogenic effects, mutation, and therapeutic implications were different in each cancer. 242 BRIP1 mutations were found across 32 cancer types. UCEC had the highest alteration (mutation and CNV) frequency. In addition, BRIP1 was a crucial oncogenic factor in OV and BRCA. BRIP1 mutation in PRAD was targetable, and FDA had approved a new drug. Moreover, Kaplan–Meier curve analysis showed that BRIP1 expression and genetic aberrations were closely related to patient survival in several cancers, indicating their potential for application as new tumor markers and therapeutic targets. The current study profiled the total BRIP1 mutation spectrum and offered an extensive molecular outlook of BRIP1 in a pan cancer analysis. And it suggested a brand-new perspective for clinical cancer therapy.

## Introduction

BRCA1 interacting helicase 1 (BRIP1) belonged to the DEAH helicase family, which bonded directly to the BRCA1^1^. It was located in human chromosome 17q23.2, containing a region of 180 kb^2^. BRIP1 consisted of 20 exons and 19 introns that encoded a protein with 1249 amino acids from 61679185 to 61863558 base pairs^3^. BRIP1 conserved helicase ATP-core binding domain which comprised eight motifs. The principal motif was the iron-sulfur (Fe-S) cluster, characterized by four conserved cysteine residues, distinguishing BRIP1 from other proteins of the DEAH helicase family^2,4^. And it was essentially required for helicase activity. Meanwhile, the N-terminal domain of BRIP1 contains seven highly conserved DEAH helicase motifs that play an important role in unfastening DNA^5–7^.

BRIP1 was expressed in both tumor and adjacent tissue (the paired adjacent normal tissue to tumor). BRIP1 harbors the function of genome integrity via the regulation of replication and homologous recombination (HR)^8,9^. DNA damage responses are crucial for genomic stability. The cells lose some normal functions when BRIP1 failed bind BRCA1. BRIP1 was initially described in Fanconi Anemia (FA)^10^. It was also called FANCJ (FA complementation group J) or BACH1. Subsequently, two convincing studies also indicated that BRIP1 was a pathogenic gene for FANCJ^11^. The following studies stated that germline alteration of BRIP1 was a risk factor for ovarian cancer (OV)^12^. For some OV patients with BRIP1 mutation, preventive surgery was increasingly recommended, especially in the high-grade serous epithelial subtype^13^. Recent investigations indicated that BRIP1 mutations were functional as a pathogenic role for breast cancer^14^. However, the role of germline BRIP1 mutations in breast cancer remained disputed^15^. In addition, BRIP1 mutations were deemed to take part in gastrointestinal (GI) cancers in some research^16^.

Recently, some targeted therapy was coming down the pike aimed at HR-deficient, such as PARP inhibitors^17^. The FDA had approved some PARP inhibitors including olaparib, niraparib and rucaparib as supportive therapy for platinum-sensitive recurrent and advanced ovarian cancer^15,18^. BRIP1 played a vital role in DNA repair via HR. Therefore, BRIP1 seemed a promising therapeutic target^14^.

Previous studies on BRIP1 mutation in cancer were only focused on individual cancer types. In the current study, we use comprehensive bioinformatic databases to analyze abnormal BRIP1 genetic spectrum. Besides, we found that BRIP1 mutation patterns were closely related to survival in several cancers. And it indicated the promising prognostic role of BRIP1 in cancers. Conclusively, our findings analyzed and summarized the potential role of BRIP1 as a promising therapeutic target in different cancer types.

## Results

### Expression and methylation analysis of BRIP1 in pan cancer

**Figure 1:**
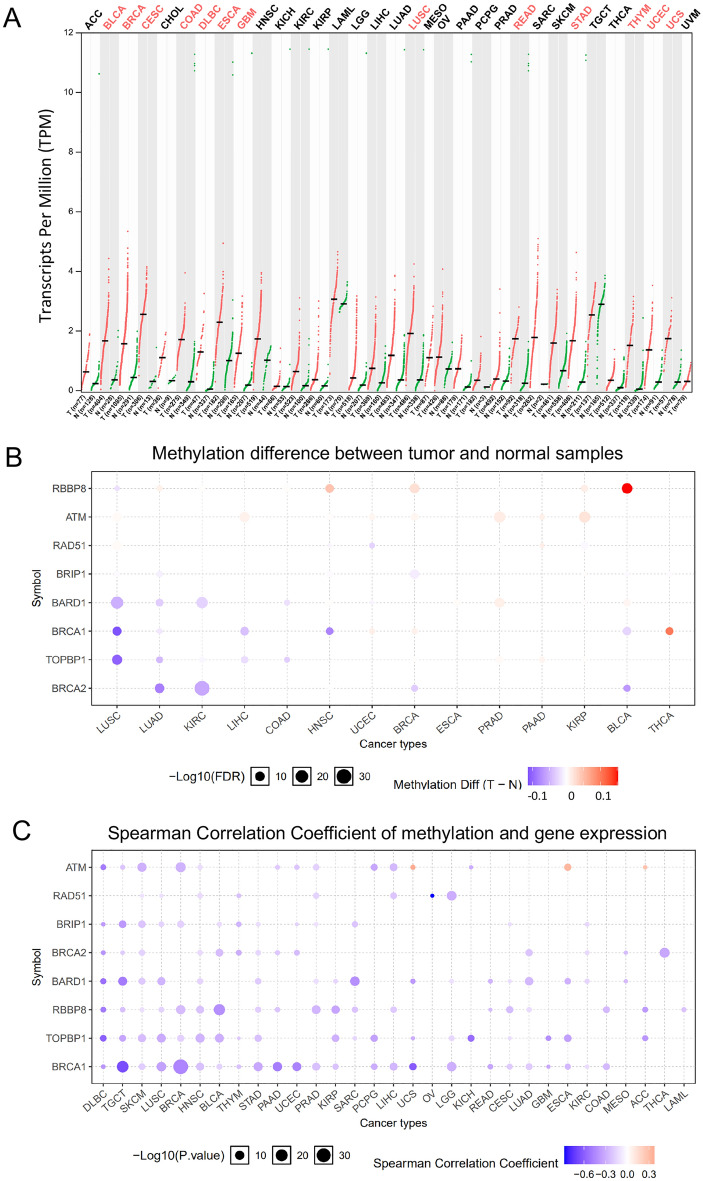
The mRNA expression and methylation levels of BRIP1 in TCGA cancer tissues. (**A**) BRIP1 mRNA expression between tumors and the matched adjacent normal tissue in various TCGA cancer types from GEPIA (*p* < 0.01 was set as statistically significant). (**B**) Bubble map showing the methylation differences of BRIP1 and downstream gene in 14 different cancer types between tumors and matching normal tissues. Cancers with red dots have higher levels of methylation, whereas those with blue dots have lower levels of methylation (T: tumor; N: the paired normal tissue). (**C**) Across cancer types, a bubble map demonstrates the relationship between methylation and the expression of BRIP1 and downstream genes. The upregulated methylation level and expression level are represented by red dots, whereas the upregulated methylation level and downregulated expression level are represented by blue dots. The size of the point in the two-bubble map shows statistical significance, with a bigger size indicating greater significance. The difference is represented by the color depth of the point; the greater the contrast, the darker the color. TPM, transcripts per million.

The abnormal expression of BRIP1 was detected in varieties of cancer types. Preceding studies of BRIP1 were limited to onefold cancer type and the relatively small number of cases. In the current work, we investigated BRIP1 expression in pan cancer. At first, we compared the mRNA expression pattern of BRIP1 between tumors and matching normal tissues among various cancer types by GEPIA2. The gene expression data of BRIP1 from TCGA and GTEx was recomputed to make them more compatible. And the recompute methods were based on the standard pipeline according to UCSC Xena Project. As the results showed (Fig. 1A), compared with the paired adjacent normal tissue to tumor, BRIP1 expression was significantly upregulated in 13 cancer types including bladder urothelial carcinoma (BLCA), breast invasive carcinoma (BRCA), cervical squamous cell carcinoma and endocervical adenocarcinoma (CESC), colon adenocarcinoma (COAD), lymphoid neoplasm diffuse large B-cell lymphoma (DLBC), esophageal carcinoma (ESCA), glioblastoma multiforme (GBM), lung squamous cell carcinoma (LUSC), rectum adenocarcinoma(READ), stomach adenocarcinoma (STAD), thymoma (THYM), uterine corpus endometrial carcinoma(UCEC) and uterine carcinosarcoma (UCS). CESC was the most increased expression in these cancer types, with 2.4 TPM (tumor) compared with 0.34 TPM (normal tissue). Interestingly, no significantly decreased expression of BRIP1 was observed among all the cancer types in this study. Furthermore, it was worth mentioning that the expression of BRIP1 was upregulated in almost gynecological tumors which indicates BRIP1 was potentially essential in gynecological oncology.

Recent studies have demonstrated that DNA methylation was closely related to the altered gene expression in malignancies. Therefore, we used the GSCALite platform to investigate the methylation status of BRIP1 and its downstream genes in various TCGA databases. First, we explored the methylation difference between tumor and normal tissues in several cancer types. The result showed that the methylation of BRIP1 was only downregulated in lung adenocarcinoma (LUAD) and BRCA while none cancer type upregulated the level methylation of BRIP1 (Fig. 1B). Then, we explored the association between BRIP1 expression and its methylation in various cancer types. We found that the expression levels of BRIP1 and downstream genes were mainly negatively related to methylation levels, with a few positive associations (Fig. 1C).

### BRIP1 alterations in different cancer types

**Figure 2:**
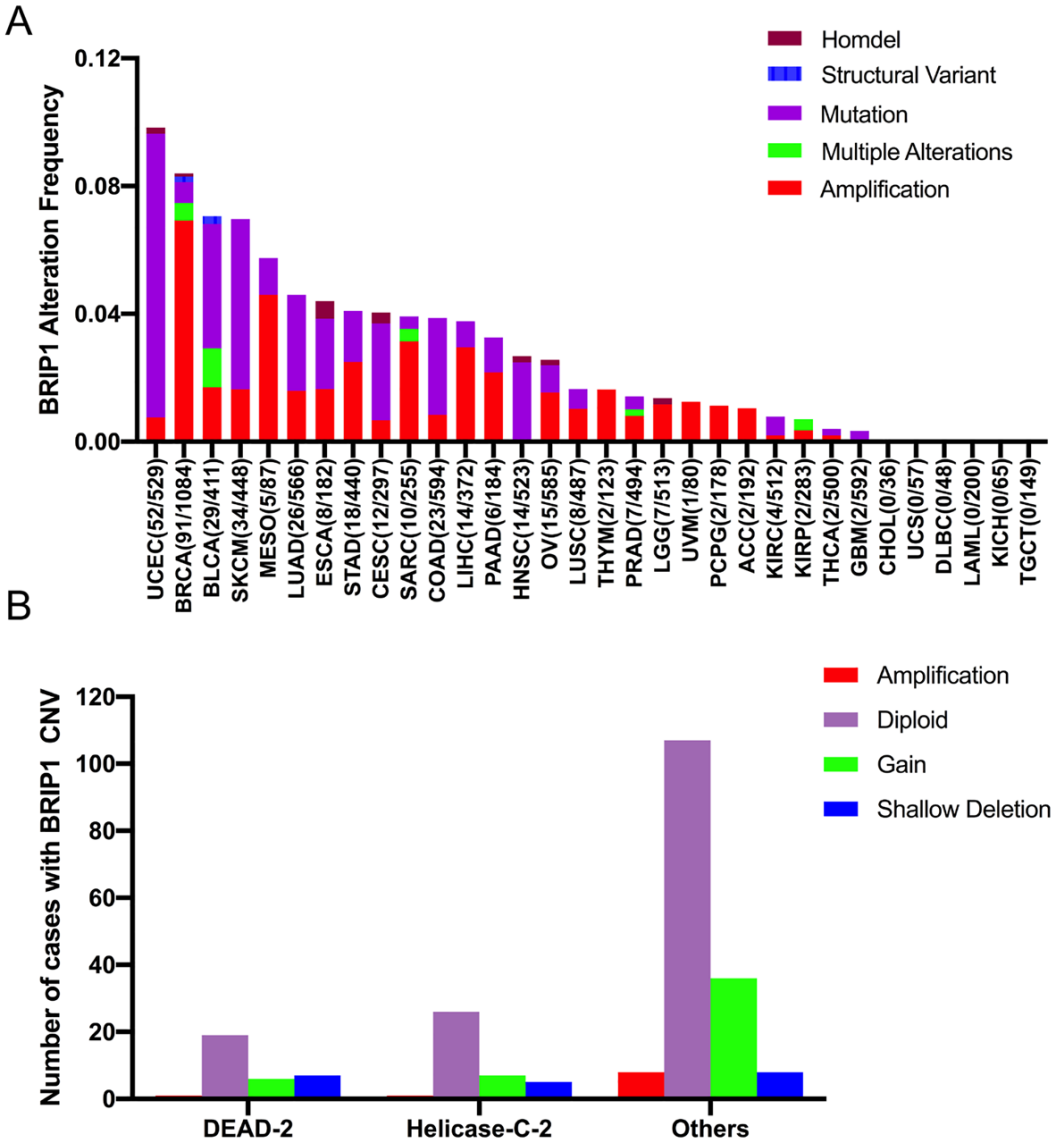
BRIP1 alteration frequency and distribution in different tumor types. (**A**) Frequency of BRIP1 alterations across different cancer types. (**B**) The distribution of the CNV types along with mutations located in various BRIP1 domains. BRIP1 domains: DEAD-2 domain (43–115 aa), Helicase-C-2 domain (264–359 aa), and other domains (481–757 aa). aa, amino acid.

The total BRIP1 alteration (mutation and copy number variants (CNV)) frequency was 2.21% in selective TCGA samples (242/10,967) containing 32 cancer types. The frequency of BRIP1 alteration presented diversity in individual cancer type, with the tumor sample number varied from 36 (CHOL) to 1084 (BRCA). UCEC (9.82%), BRCA (8.39%) and BLCA (7.05%) were the cancer types ranked ahead with the higher BRIP1 alteration. Among the five types (Amplification, Multiple alterations, Mutation, Structural variant, and Homozygously deleted) of BRIP1 alteration, tumors with dominant BRIP1 amplification included BRCA (6.91%), MESO (4.59%), SARC (3.13%), LIHC (2.95%), and STAD (2.50%). Structural variant only occurred in BRCA (0.145%) and BLCA (0.243%). Homozygously deleted was occasionally found in ESCA (0.54%), CESC (0.33%), LGG (0.19%), HNSC (0.19%), UCEC (0.18%). Moreover, several cancer types mainly had BRIP1 mutations but relatively few multiple alterations, such as UCEC, SKCM, BLCA, LUAD, and CESC (8.88 vs. 0%, 5.32 vs. 0%, 3.89 vs. 1.21%, 3.0 vs. 0%, and 3.03 vs. 0%, respectively). What’s more, cancer types including CHOL, USC, DLBC, LAML, KICH, and TGCT, did not occur any BRIP alterations (mutation and CNVs) (Fig. 2A).

We mentioned that there were three functional domains in BRIP1 protein structure including the Helicase-C-2 domain (43–115 aa), DEAD-2 domain (172–248 aa) and the other domain. The current study also analyzed the correlation between mutation location and CNV occurrence. As a consequence, 231 cases with BRIP1 somatic mutations accompanied with CNVs were found in 32 cancer types. The most common domains with BRIP1 mutations were the other domain (159 samples), followed by the Helicase-C-2 domain (26 samples), and the DEAD-2 domain (21 samples) (Fig. 2B).

### BRIP1 somatic mutation patterns across cancer types

**Figure 3:**
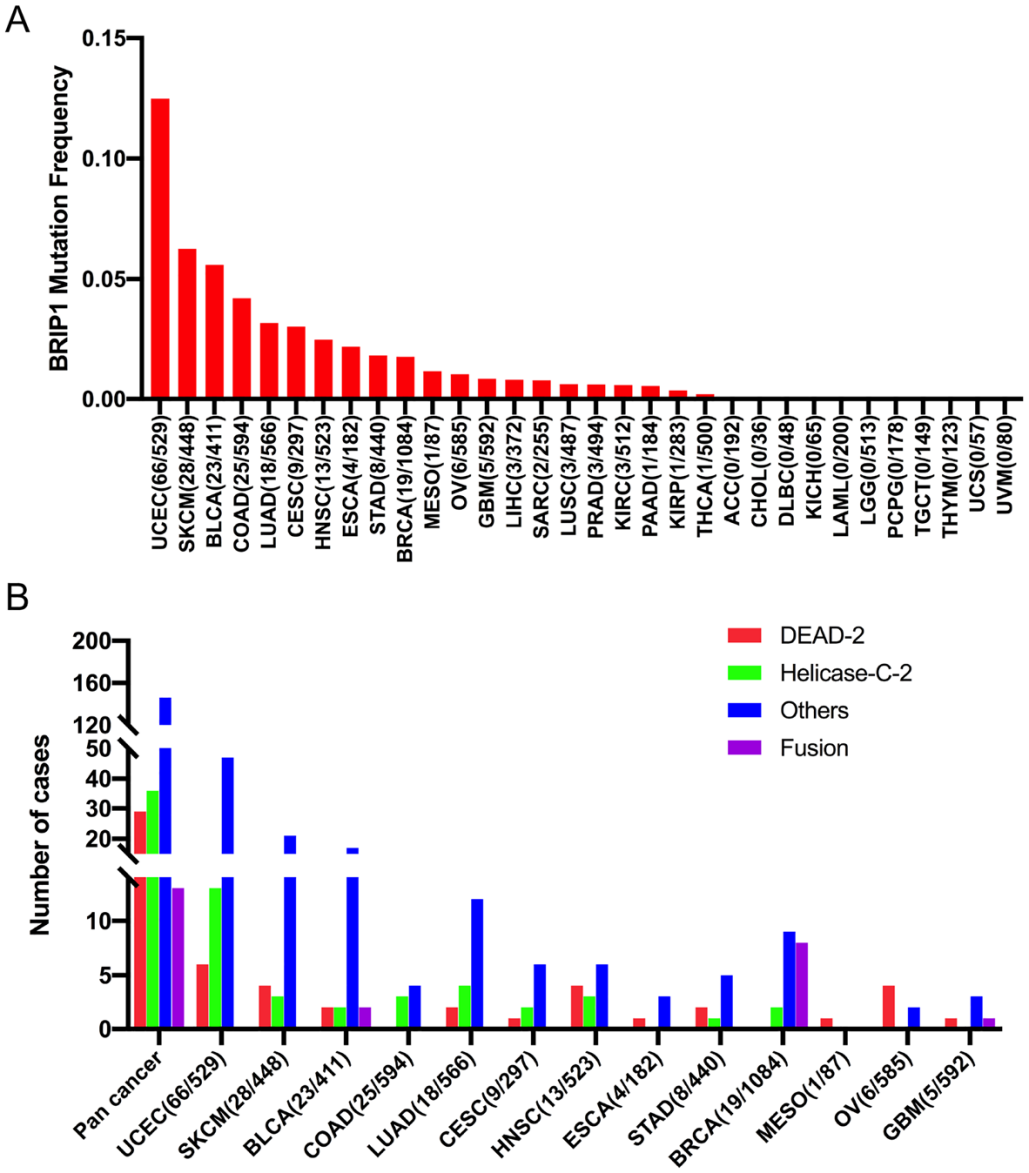
The mutation frequency of BRIP1 in various TCGA cancers and protein functional domains. (**A**) The BRIP1 mutation frequency in 32 TCGA cancer types. (**B**) The mutation location of BRIP1 in BRIP1 protein domains in pan cancer and in the top 13 cancer types.

In all tumor samples, the total mutation frequency of BRIP1 was 2.18% (242/11,066) (Supplementary Table S1). UCEC, SKCM, BLCA, COAD, and LUAD were the most common malignancies with BRIP1 mutation, with frequencies of 12.47%, 6.25%, 5.59%, 4.20%, and 3.18%, respectively. On the contrary, ACC, CHOL, DLBC, KICH, LAML, LGG, PCPG, TGCT, THYM, UCS, and UVM showed nearly no BRIP1 mutations. Some cancer types with insufficient samples may not represent the BRIP1 mutation profile (Fig. 3A).

Subsequently, 242 BRIP1 somatic mutations across 32 cancers were further stratified according to their locations in functional domains. The most common domains with BRIP mutations were the other domain (146 samples). The second was Helicase-C-2 domain (36 samples). And the least was the DEAD-2 domain (29 samples). Besides, fusions (13 samples) were also found. Moreover, the distribution diagram of BRIP1 mutations in these three functional domains varied greatly among different cancers. 14 presentative cancer types with more BRIP1 mutations were enrolled to generate the distribution diagram in pan cancer. We found that mutations in UCEC, SKCM, BLCA, and LUAD were most commonly located in the other domain, the functions of which were rarely known, especially for SKCM, which amounted to 75% of all somatic mutations. Mutations of OV and HNSC were commonly located in DEAD-2 or Helicase-C-2 domain. UCEC harbored the most mutation in the Helicase-C-2 domain and DEAD-2 domain across the cancer types. Notably, fusion occurred mostly in BRCA and BLCA (Fig. 3B, Supplementary Table S2).

**Figure 4:**
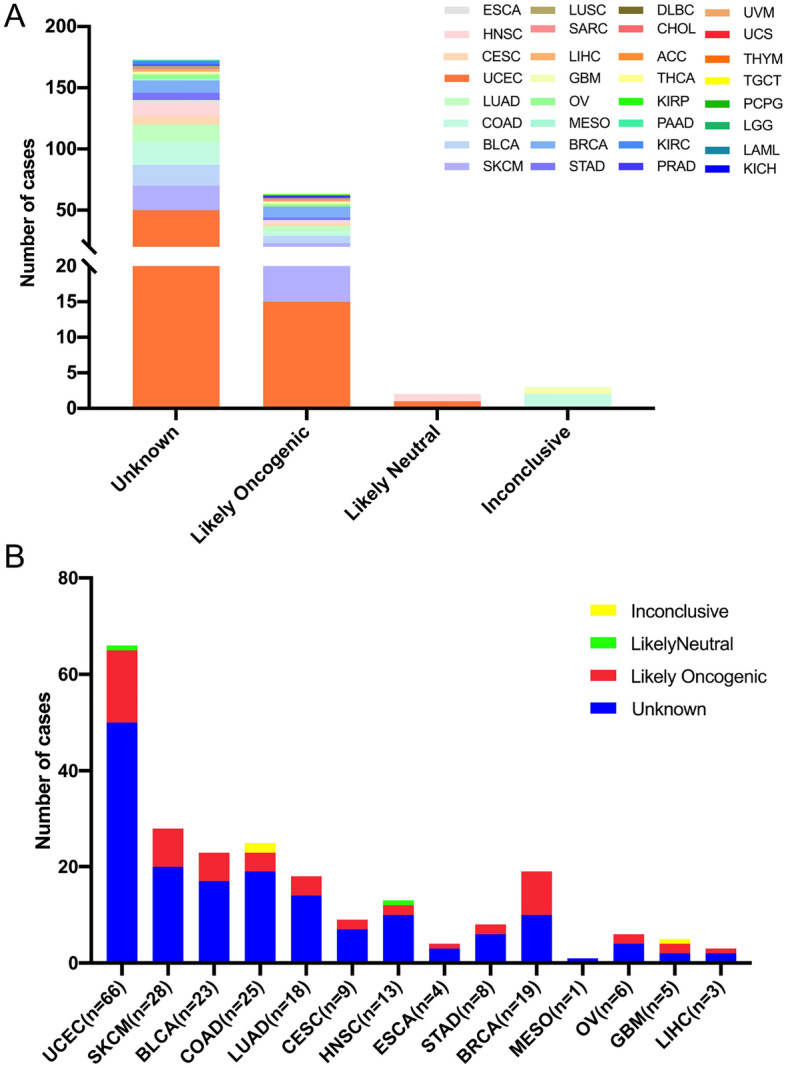
The distribution of BRIP1 mutations based on functional impacts. (**A**) The classification of BRIP1 mutations according to functional effects on various cancers collectively. (**B**) Distribution of BRIP1 mutations by functional impact class in the top 14 cancer types.

Next, we further classified the 242 mutations into four categories based on oncogenic effects and predictive significance. The four classifications were unknown (173 mutations), likely oncogenic (64 mutations), inconclusive (3 mutations), and likely neutral (2 mutations). Nearly two-thirds of these somatic mutations belonged to the unknown class, suggesting the challenges as well as value of deeper mechanistic researches in exploring their specific functions (Fig. 4A, Supplementary Table S1). In addition, likely oncogenic mutations mainly occurred in UCEC, BRCA, SKCM, and BLCA. The highest proportion of likely oncogenic mutation was BRCA in all cancer types (Fig. 4B). These results highlighted the importance of BRIP1 mutations in oncogenesis but the deep mechanism and evidence were lacked, indicating the potential of BRIP1 in tumor therapy.

**Figure 5:**
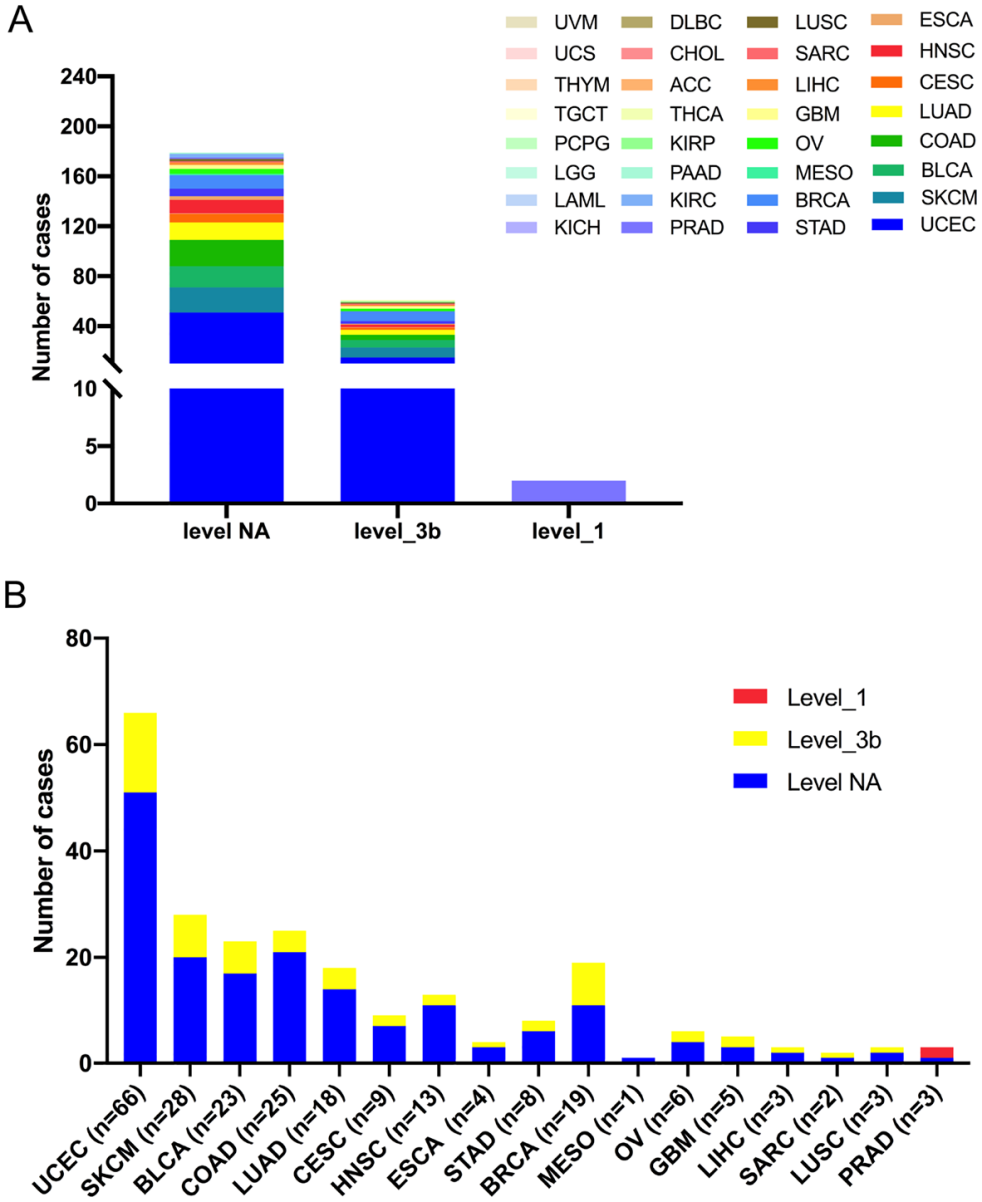
The classification of BRIP1 mutations according to potential effects of targeted therapy. (**A**) Based on the clinical therapy implications as annotated in OncoKB, BRIP1 mutation categorization is performed. (**B**) Distribution of BRIP1 mutations by therapy implications in pan cancer and in the top 17 cancer types.

Then, the 242 BRIP1 somatic mutations were divided into three levels based on the clinical targeted therapy implications defined using OncoKB from cBioPortal (Fig. 5A, Supplementary Table S1): level NA (179 mutations), level 3B (61 mutations), and level 1 (2 mutations). Most BRIP1 mutations were belonged to the NA class without targeted therapy implications. Besides, all level 1 mutations were observed in PRAD. This meant BRIP was a predictive biomarker for some FDA-approved drugs. In addition, mutations belonged to level 3b accounted for nearly a half of BRCA (8/19) and SKCM (8/20) (Fig. 5B).

### BRIP1 CNVs in different cancer types

**Figure 6:**
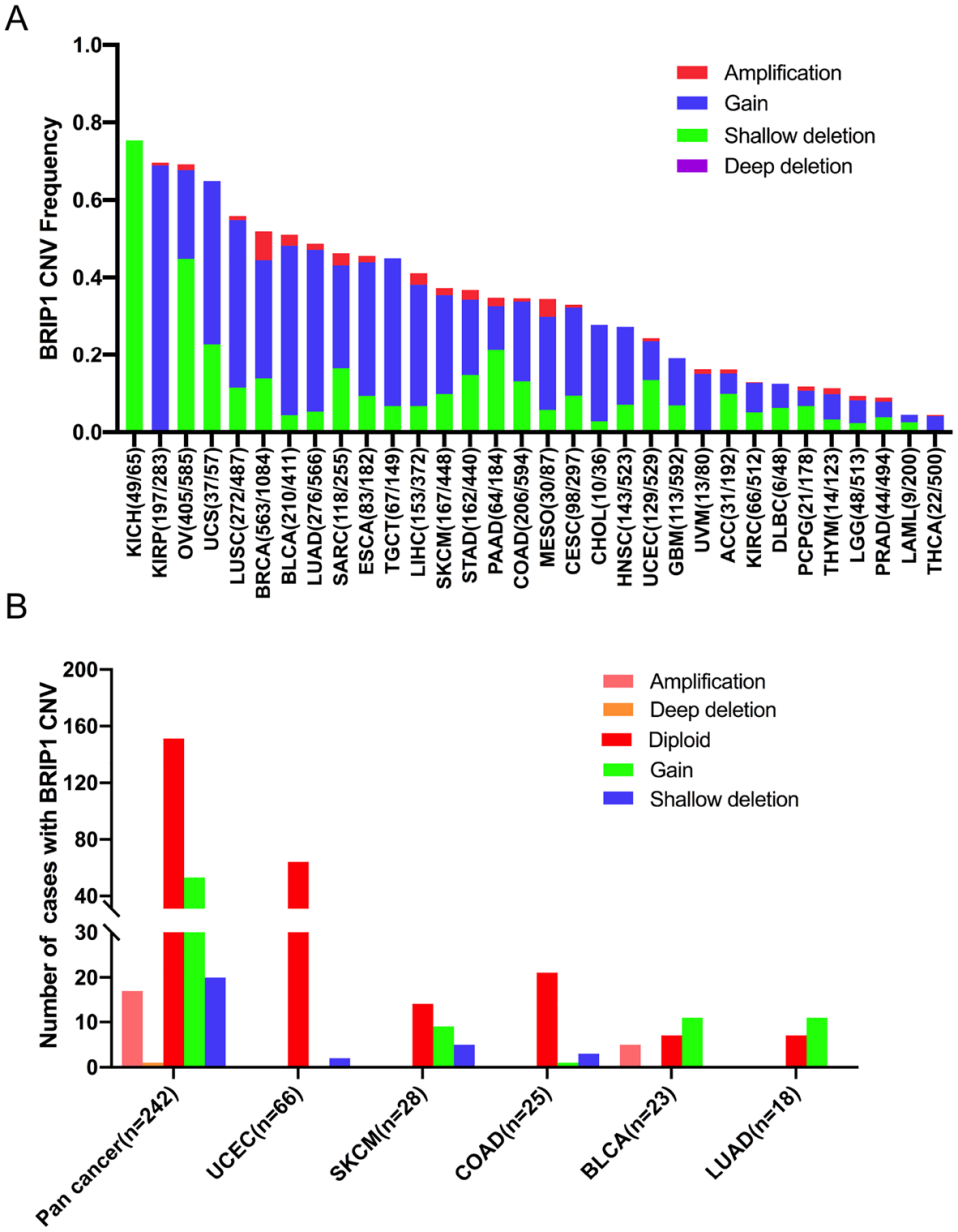
The distribution of BRIP1 CNV among different cancer types. (**A**) The CNV frequency of BRIP1 in 32 tumor types. (**B**) The CNV distribution of BRIP1 in pan cancer and in the top 5 tumor types for the samples with BRIP1 mutations simultaneously.

CNVs were closely related to tumor progression. It played a vital role in oncogene activation and tumor suppressor gene inactivation, ultimately leading to the occurrence of tumors. In this section, we used the ciBioportal to explore BRIP1 CNVs in different cancer. The total BRIP1 CNV frequency was 34.88% (3826 of 10,967 samples) in pan cancer and showed remarkable diversity for individual cancer ranging from 4.4 to 75.38%. KICH (75.38%), KIRP (69.61%), OV (69.23%), UCS (64.91%), and LUSC (55.85%) were the most common tumors with BRIP1 CNVs. Conversely, PRAD (8.90%), LAML (4.50%), and THCA (4.41%) exhibited relatively low frequencies of BRIP1 CNVs. The CNVs were classified into four groups: gain, shallow deletion, amplification, and deep deletion. Most of the CNVs of BRIP1 belonged to the gain group (2445 samples). The second was shallow deletion (1175 samples), followed by amplification (198 samples), and deep deletion (8 samples). In KIRP, nearly 90% CNVs of BRIP1 belonged to gain. BRCA harbored the most CNVs of amplification. The CNVs of shallow deletion account for the most proportion of KICH and OV (Fig. 6A). Then, we explored the correlation between BRIP1 CNVs and BRIP1 expression. They were compared across TCGA cancer types, and the results showed that there was a positive correlation between mRNA expression and CNVs of BRIP1 in pan cancer (r = 0.2059, p < 0.0001) (Supplementary Fig. S1).

In addition, the correlation between BRIP1 somatic mutations and CNVs was analyzed. We found that among the 242 samples with BRIP1 somatic mutations, 109 also harbored BRIP1 CNVs, of which 17 harbored amplifications, one harbored deep deletion, 53 harbored gain, and 20 harbored shallow deletion. Specifically, BLCA harbors the highest number of amplifications. BLCA and LUAD were the cancer types with the highest number of gain (Fig. 6B).

### BRIP1 alterations and patient survival

**Figure 7:**
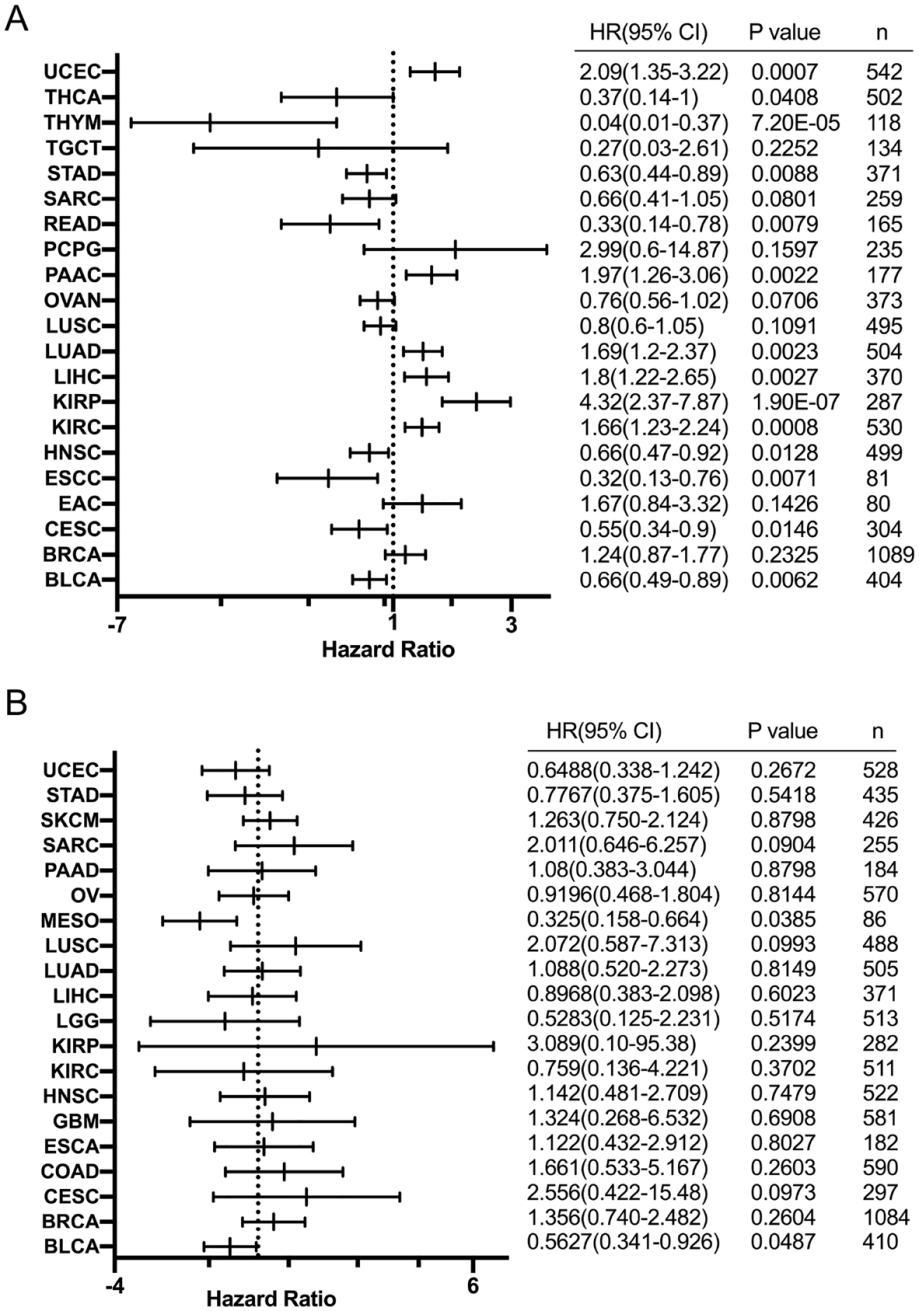
Correlation between BRIP1 alteration and patient prognosis. (**A**) The relationship between BRIP expression and overall survival. (**B**) The relationship between BRIP alteration and patient overall survival.

At last, we analyzed the prognostic significance of BRIP1 in each cancer. The results manifested that decreased BRIP1 expression was related to poor overall survival (OS) in BLCA, CESC, ESCC, HNSC, READ, STAD, THYM and THCA. While it was increased BRIP1 expression that was associated with poor OS in KIRC, KIRP, LIHC, LUAD, PAAC and UCEC (Fig. 7A). In addition, survival association analysis between BRIP1 mRNA expression and patient relapse free survival (RFS) in each cancer type showed that in KIRC, OV, and STAD, decreased BRIP1 expression was associated with poor RFS, while among patients with KIRP, LIHC, LUAD, PAAC, SARC or THCA increased BRIP1 expression was associated with poor RFS (Supplementary Fig. S2).

Furthermore, the association of survival regarding the alteration status of BRIP1 was also analyzed in individual cancer types. The result showed that BRIP1 alteration was associated with a better prognosis in BLCA and MESO. And we have not found the association between BRIP1 alteration and worse prognosis in pan cancer (Fig. 7B).

## Discussion

In the current study, we profiled the features of BRIP1 in 32 TCGA cancer types. We also analyzed the important therapeutic and clinical importance of BRIP1. The results showed that expression, methylation, and alteration of BRIP1 varied greatly from each cancer by analyzing large comprehensive datasets with over 10,000 tumor samples. BRIP1 expression was significantly upregulated in 13 tumors, especially in CESC. The total alteration frequency of BRIP1 across all tumor types was relatively low (2.21%), and mutation took up a major portion in most cancer types.

Some findings had stated that BRIP1 was a crucial oncogenic gene in ovarian cancer^15,19,20^. In this pan cancer analysis, the alteration of BRIP1 was 1.53% in OV, and the mutation was 1.02%. The mutations of BRIP1 were commonly located in DEAD-2. Similarly, previous studies stated mutations of BRIP1 were ~ 2% of OC patients^21^. Several recent data suggested that the BRIP1 genes may be the most important OV predisposition genes^13,22^. The approximated accumulated OV risk is 5.8 for BRIP1 (by age 80) mutation carriers^13^. In our study, there were 6 mutations in OV and 2 of them were classified in to likely oncogenic. We used the OncoKB to explore the targeted therapy implications. Two mutations of BRIP1 in OV were belonged to level 3b, which meant that FDA approved some drugs which used BRIP1 as therapeutic biomarkers in oncology treatments. Previous studies indicated patients with germline mutations in BRIP1, could likely benefit from therapy with PARP inhibitors^23–25^. Another clinical investigation indicated using the treatment of a platinum agent/ PARP inhibitors combination in ovarian cancers might bring a better prognosis^20^. What’s more, recent investigations indicated Olaparib monotherapy had the potential in treating endometrial cancers with BRIP1 mutations^26^.

In addition, previous publications indicated BRIP1 could plausibly play an oncogenic role in BRCA^12,27^. BRIP1 is involved in DNA repair which was critical process in the occurrence of breast cancer^28^. In our study, nearly half mutation of BRIP1 in BRCA was classified as likely oncogenic which meant BRIP1 mutation plays a crucial role in BRCA. Moreover, the mutation and expression of BRIP1 significantly influenced the prognosis in BRCA patients. An Omani study showed that BRIP1 overexpression is correlated with clinical features and survival outcomes of BRCA patients^28^. Subsequently, the following studies found BRIP1 promoting breast cancer cell invasion and migration^12,29^. Therefore, BRIP1 might be a significant gene that could serve as biomarkers and/or targets to guide the design of appropriate BRCA targeted therapies.

Frequent BRIP1 mutations had been observed in UCEC (12.47%). It indicated that BRIP1 had great potential for targeted therapy. In our study, UCEC had the highest frequency of BRIP1 alteration, which was driven by a high proportion of mutations. One-third of frequently mutated positions in UCEC were classified as likely oncogenic. All of them were classified as the level 3b class, which represented they might have standard care or potential investigational drug. However, mutation as a therapeutic target of BRIP1 inhibitors had exhibited disappointing clinical outcomes. The FDA has yet to approve any targeted therapeutic drug of BRIP1 for UCEC. So, it is pivotal to develop targeted drugs against BRIP1 in UCEC.

What was noteworthy was that PRAD was the only cancer type in which FDA had approved inhibitors targeted BRIP1 mutation. In our study, the BRIP1 mutation frequency of PRAD was 0.61% whereas most of them were likely oncogenic. Loss of BRIP1 function is associated with familial prostate cancer^30–32^. Some evidence also indicated that BRIP1 mutation was carcinogenic in familial and young onset prostate cancer^33^. BRIP1 mutation was also associated with gastrointestinal tumors^34–36^. Recent publications evaluated the role of the BRIP1 variant in hereditary colorectal cancer. Besides, it described three germline BRIP1 variants in three unrelated families^34^. Subsequent studies deeply confirmed that BRIP1 mutation increased the risk of colorectal cancer^34,37^. In the current study, twenty-five mutations of BRIP1 were identified among 594 patients. Four mutations were considered likely oncogenic while most of the mutation functions were unknown which meant more efforts need to explore the role of BRIP1 in the process of COAD.

In the current study, we explored BRIP1 expression, methylation, alteration, and their clinical associations in 32 cancer types. However, there are still several limitations that should be mentioned in this study. First, although this is a pan-can analysis, some tumors are not representative because of the small sample size. Moreover, this study lacked an in-depth analysis of each tumor. Furthermore, BRIP1 alteration was rare in some tumor types. And this low alteration frequency could make our analysis challenging. Besides, the BRIP1 expression related OS also lacked 12 cancer types. Therefore, more cases were needed to verify the function of predicting the prognosis. Subsequent cell function experiments also need to be further refined to verify our findings.

## Conclusions

In conclusion, the current study provided the first comprehensive pan cancer profile of BRIP1 abnormal expression, methylation, alteration, and their therapeutic and prognostic implications, covering 10,967 tumor samples across 32 cancer types. Several BRIP1 alterations were involved in the occurrence of tumors and participated in targeted treatment. BRIP1 alterations and expression were correlated with patient prognosis in some cancer types. The results of this analysis provided important new insights into the dysregulation of BRIP1 in tumor biology and identified potential therapeutic targets and prognostic indicators for tumors.

## Materials and methods

### Data acquisition and reanalysis using different bioinformatics tools

The BRIP1 mRNA expression profiles were compared between malignant and matched tissues via the GEPIA2 database. The data about BRIP1 mRNA expression were downloaded from TCGA. Next, different methylation of BRIP1 and downstream genes were investigated. Meanwhile, we also explored the association between methylation and the expression of BIPP1 and downstream genes in different cancers.cBioPortal, an open comprehensive database, which contained large-scale tumor genomics data, providing tumor genomics and clinical data for users. In the current study, we comprehensively investigated BRIP1 mutation, using the “TCGA pan cancer atlas studies” containing 10,967 samples from 32 cancer types (Supplementary Table S3). The BRIP1 mRNA expression data were generated by normalizing values with the reference population of all samples independent of sample diploid status, termed as NormalizeExpressionLevels_allsampleref.py, and was log10 transformed after downloaded from cBioportal. For the BRIP1 CNV data, the log ratio value represents: 2 = amplification; 1 = gain; 0 = diploid; − 1 = shallow deletion; and − 2 = deep deletion.

Moreover, we analyzed the association between BRIP1 alteration and patient survival by cBioportal database. Kaplan–Meier Plotter was used to investigate the association between BRIP1 expression and patient survival. Besides, we drew the forest plot to describe the hazard ratio for pan cancer survival analyses.

### Statistical analyses

The statistical analyses were analyzed with SPSS 22.0 software (IBM Analytics, United States). Student’s t-test, Cox regression analysis, and linear regression analysis were used to analyze the results. *p* < 0.05 was set as statistically significant. Online analysis websites of GEPIA2 (http://gepia.cancer-pku.cn/), cBioportal (http://cbioportal.org/), GSCA Lite (http://bioinfo.life.hust.edu.cn/web/GSCALite/), and Kaplan–Meier Plotter (http://kmplot.com/) were also used.

## Supplementary Information

Supplementary Figures.

Supplementary Table S1.

Supplementary Table S2.

Supplementary Table S3.

## Data Availability

The datasets analysed in this study are available in the cBioportal (http://cbioportal.org/) and Kaplan–Meier Plotter (http://kmplot.com/).

## Abbreviations

BRIP1: BRCA1 interacting helicase 1
CNV: Copy number variant
HR: Homologous recombination
GTEx: Genotype-Tissue Expression
OS: Overall survival
RFS: Relapse free survival
