# Supplementary material for: Spectrum of BRCA1 interacting helicase 1 aberrations and potential prognostic and therapeutic implication: a pan cancer analysis
Source: Sci Rep. 2023 Mar 17;13:4435. doi: 10.1038/s41598-023-31109-6 (PMC10023799; doi:10.1038/s41598-023-31109-6)
Supplement: Supplementary file 1 — Supplementary Figures. [file 41598_2023_31109_MOESM1_ESM.docx]

**
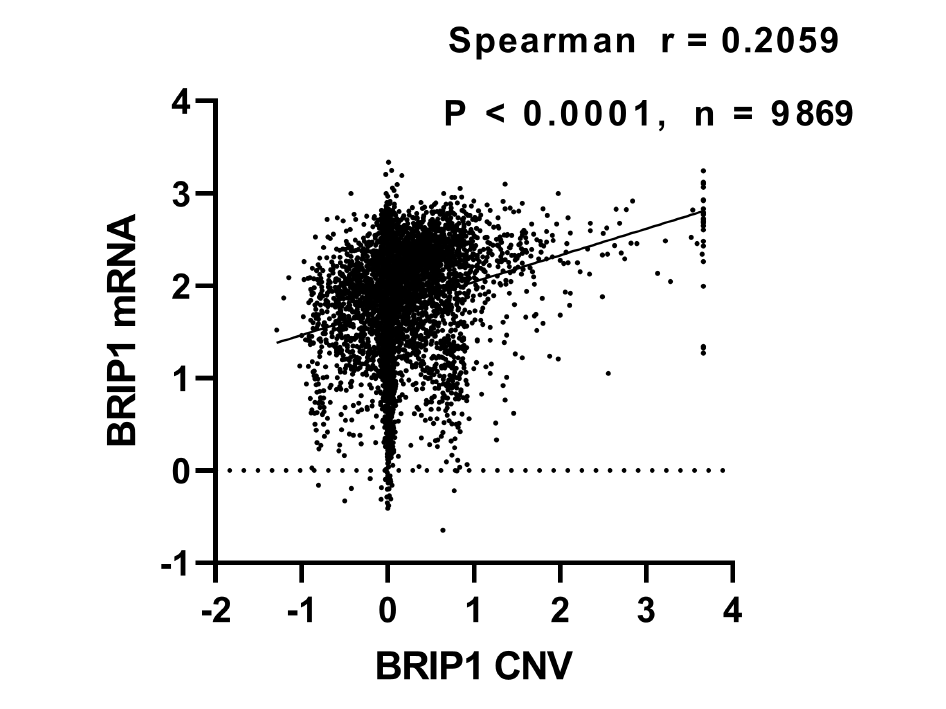
**

**Supplementary Figure S1. The mRNA expression of BRIP1 correlates with the linear copy number values of BRIP1 in various cancers.**

**
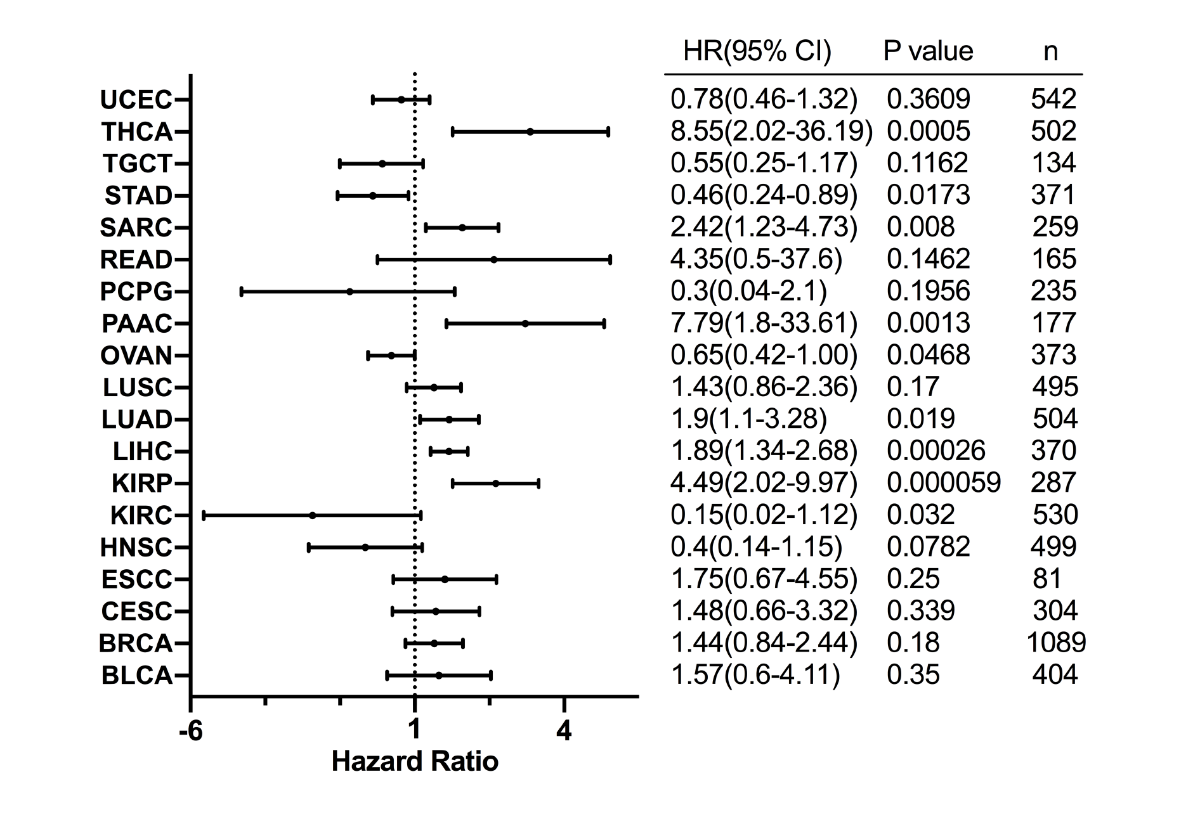
**

**Supplementary Figure S2. Correlation between BRIP1 expression and relapse free survival (RFS) in different cancers.**
