# Supplementary material for: Spectrum of BRCA1 interacting helicase 1 aberrations and potential prognostic and therapeutic implication: a pan cancer analysis
Source: Sci Rep. 2023 Mar 17;13:4435. doi: 10.1038/s41598-023-31109-6 (PMC10023799; doi:10.1038/s41598-023-31109-6)
Supplement: Supplementary file 4 — Supplementary Table S3. [file 41598_2023_31109_MOESM4_ESM.docx]

**Supplementary Table S3.** Summary of 32 TCGA tumor types and sample sizes.

| TCGA Cancer Abbreviation | TCGA Cancer Type | Sample Number |
| --- | --- | --- |
| ACC | Adrenocortical carcinoma | 92 |
| BLCA | Bladder urothelial carcinoma | 411 |
| BRCA | Breast invasive carcinoma | 1084 |
| CESC | Cervical squamous cell carcinoma and endocervical adenocarcinoma | 297 |
| CHOL | Cholangiocarcinoma | 36 |
| COADREAD | Colon adenocarcinoma /Rectum adenocarcinoma | 594 |
| DLBC | Lymphoid neoplasm diffuse large B-cell lymphoma | 48 |
| ESCA | Esophageal carcinoma | 182 |
| GBM | Glioblastoma multiforme | 592 |
| HNSC | Head and Neck squamous cell carcinoma | 523 |
| KICH | Kidney chromophobe | 65 |
| KIRC | Kidney renal clear cell carcinoma | 512 |
| KIRP | Kidney renal papillary cell carcinoma | 283 |
| LAML | Acute myeloid leukemia | 200 |
| LGG | Brain lower grade glioma | 513 |
| LIHC | Liver hepatocellular carcinoma | 372 |
| LUAD | Lung adenocarcinoma | 566 |
| LUSC | Lung squamous cell carcinoma | 487 |
| MESO | Mesothelioma | 87 |
| OV | Ovarian serous cystadenocarcinoma | 585 |
| PAAD | Pancreatic adenocarcinoma | 184 |
| PCPG | Pheochromocytoma and Paraganglioma | 178 |
| PRAD | Prostate adenocarcinoma | 494 |
| SARC | Sarcoma | 255 |
| SKCM | Skin cutaneous melanoma | 448 |
| STAD | Stomach adenocarcinoma | 440 |
| TGCT | Testicular germ cell tumors | 149 |
| THCA | Thyroid carcinoma | 500 |
| THYM | Thymoma | 123 |
| UCEC | Uterine corpus endometrial carcinoma | 529 |
| UCS | Uterine carcinosarcoma | 57 |
| UVM | Uveal Melanoma | 80 |
